# Targeting pyroptosis as a preventive and therapeutic approach for stroke

**DOI:** 10.1038/s41420-023-01440-y

**Published:** 2023-05-10

**Authors:** Junpeng Long, Yang Sun, Shasha Liu, Songwei Yang, Chen Chen, Zhao Zhang, Shifeng Chu, Yantao Yang, Gang Pei, Meiyu Lin, Qian Yan, Jiao Yao, Yuting Lin, Fan Yi, Lei Meng, Yong Tan, Qidi Ai, Naihong Chen

**Affiliations:** 1 Hunan Engineering Technology Center of Standardization and Function of Chinese Herbal Decoction Pieces, College of Pharmacy, Hunan University of Chinese Medicine, Changsha, P. R. China; 2 Department of Pharmacy, Changsha Hospital for Matemal & Child Health Care, Changsha, P. R. China; 3 Department of Pharmacy, The First Hospital of Lanzhou University, Lanzhou, P. R. China; 4 State Key Laboratory of Bioactive Substances and Functions of Natural Medicines, Institute of Materia Medica & Neuroscience Center, Chinese Academy of Medical Sciences and Peking Union Medical College, Beijing, P. R. China; 5 Key Laboratory of Cosmetic, China National Light Industry, Beijing Technology and Business University, Beijing, P. R. China; 6 Department of Nephrology, Xiangtan Central Hospital, Xiangtan, P. R. China

**Keywords:** Cell death in the nervous system, Stroke

## Abstract

Stroke has caused tremendous social stress worldwide, yet despite decades of research and development of new stroke drugs, most have failed and rt-PA (Recombinant tissue plasminogen activator) is still the accepted treatment for ischemic stroke. the complexity of the stroke mechanism has led to unsatisfactory efficacy of most drugs in clinical trials, indicating that there are still many gaps in our understanding of stroke. Pyroptosis is a programmed cell death (PCD) with inflammatory properties and are thought to be closely associated with stroke. Pyroptosis is regulated by the GSDMD of the gasdermin family, which when cleaved by Caspase-1/Caspase-11 into N-GSDMD with pore-forming activity can bind to the plasma membrane to form small 10–20 nm pores, which would allow the release of inflammatory factors IL-18 and IL-1β before cell rupture, greatly exacerbating the inflammatory response. The pyroptosis occurs mainly in the border zone of cerebral infarction, and glial cells, neuronal cells and brain microvascular endothelial cells (BMECs) all undergo pyroptosis after stroke, which largely exacerbates the breakdown of the blood-brain barrier (BBB) and thus aggravates brain injury. Therefore, pyroptosis may be a good direction for the treatment of stroke. In this review, we focus on the latest mechanisms of action of pyroptosis and the process by which pyroptosis regulates stroke development. We also suggest potential therapeutic stroke drugs that target the pyroptosis pathway, providing additional therapeutic strategies for the clinical management of stroke.

**Figure:**
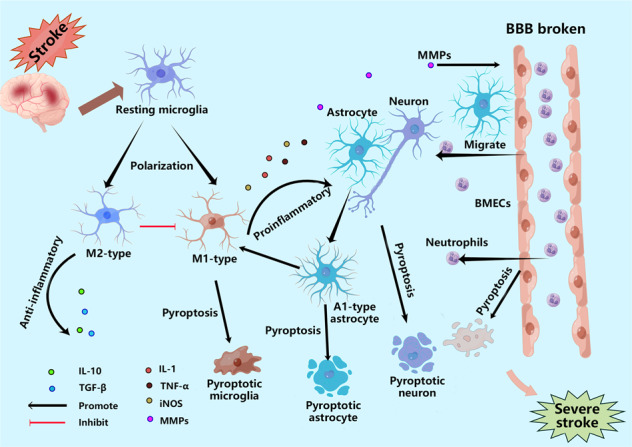
**The role of pyroptosis after stroke**. After stroke, microglia first rush to the damaged area and polarize into M1 and M2 types. Under the influence of various stimuli, microglia undergo pyroptosis, release pro-inflammatory factors, and are converted to the M1 type; astrocytes and neuronal cells also undergo pyroptosis under the stimulation of various pro-inflammatory factors, leading to astrocyte death due to increased osmotic pressure in the membrane, resulting in water absorption and swelling until rupture. BMECs, the main structural component of the BBB, also undergo pyroptosis when stimulated by pro-inflammatory factors released from microglia and astrocytes, leading to the destruction of the structural integrity of the BBB, ultimately causing more severe brain damage. In addition, GSDMD in neutrophils mainly mediate the release of NETs rather than pyroptosis, which also aggravates brain injury. IL-10=interleukin-10; TGF-β = transforming growth factor-β; IL-18=interleukin-18; IL-1β = interleukin-1β; TNF-α = tumor necrosis factor-α; iNOS=induced nitrogen monoxide synthase; MMPs=Matrix metalloproteinases; GSDMD = gasdermin D; BMECs=brain microvascular endothelial cells; BBB = blood-brain barrier.

**The role of pyroptosis after stroke**. After stroke, microglia first rush to the damaged area and polarize into M1 and M2 types. Under the influence of various stimuli, microglia undergo pyroptosis, release pro-inflammatory factors, and are converted to the M1 type; astrocytes and neuronal cells also undergo pyroptosis under the stimulation of various pro-inflammatory factors, leading to astrocyte death due to increased osmotic pressure in the membrane, resulting in water absorption and swelling until rupture. BMECs, the main structural component of the BBB, also undergo pyroptosis when stimulated by pro-inflammatory factors released from microglia and astrocytes, leading to the destruction of the structural integrity of the BBB, ultimately causing more severe brain damage. In addition, GSDMD in neutrophils mainly mediate the release of NETs rather than pyroptosis, which also aggravates brain injury. IL-10=interleukin-10; TGF-β = transforming growth factor-β; IL-18=interleukin-18; IL-1β = interleukin-1β; TNF-α = tumor necrosis factor-α; iNOS=induced nitrogen monoxide synthase; MMPs=Matrix metalloproteinases; GSDMD = gasdermin D; BMECs=brain microvascular endothelial cells; BBB = blood-brain barrier.

## Facts

- Pyroptosis is a programmed cell death that occurs with inflammation and is closely associated with neuroinflammation in the early stages of stroke.
- Crosstalk between the three PCDs (programmed cell death) – pyroptosis, apoptosis, and necroptosis – is involved in the stroke onset process. Stroke may induce PANoptosis (pyroptosis, apoptosis, and necroptosis) during periods of viral invasion.
- Stroke can trigger pyroptosis of microglia, astrocytes, neurons and BMECs (brain microvascular endothelial cells), which is mainly regulated by GSDMD (gasdermin D). However, GSDMD induces NETs (neutrophil extracellular traps) but not pyroptosis in neutrophils after stroke.
- Targeted inhibition of the pyroptosis pathway is a potential therapeutic approach for stroke.

## Open questions

- What is the exact mechanism for PANoptosis to regulate cell death after stroke?
- What role does the gasdermin family play in stroke?
- How to effectively reduce pyroptosis in the clinical treatment of stroke?

## Introduction

Stroke is a life-threatening neurodegenerative disease that primarily occurs in the elderly population. Stroke can be roughly divided into hemorrhagic stroke and ischemic stroke, and the most common type in clinical practice is the ischemic. According to incomplete statistics, 800,000 strokes occur in the United States each year, 80% of which are ischemic [1, 2]. Ischemic stroke is caused by blockage of arteries in the brain, resulting in insufficient blood supply to the brain, causing brain tissue hypoxia and necrosis. Therefore, the most common treatment for ischemic stroke is thrombolytic therapy. Currently, rt-PA (Recombinant tissue plasminogen activator) is one of the most commonly used thrombolytic drugs, which exerts a good therapeutic effect within 3–4.5 h of onset in patients with ischemic stroke, but may cause ischemia/reperfusion (I/R) injury once the appropriate therapeutic time window is exceeded [3]. In addition to thrombolytic drugs, mechanical thrombectomy or anti-atherosclerotic drugs can be used to treat ischemic strokes, although many stroke patients still have poor postoperative outcomes. After a ischemic stroke, brain tissue is damaged by the stressful stimulus of hypoxia, which induces an immune-mediated inflammatory cascade. Thus, in recent years, neuroprotection has been considered as an adjunctive therapy for the treatment of stroke.

The pathogenesis of stroke is complex, and its onset is rapid. The most intense phase of a stroke is 1–2 weeks after its onset, during which multiple cells are involved. The first cells to be recruited in the area of brain damage are microglia, and the resting microglia differentiate into M1 and M2 types after stimulation. M1-type polarized microglia release large amounts of pro-inflammatory factors that induce neuroinflammation, which triggers neuronal cell and astrocyte damage, exacerbating the inflammatory response [4]. Neutrophils residing within capillaries, once they sense a large number of cells in an inflammatory response, migrate directionally to the stroke area to engulf these cells, further exacerbating brain damage [5]. Brain microvascular endothelial cells (BMECs) are among the main components of the blood-brain barrier (BBB) and are closely related to its integrity. During stroke, the biological function of BMECs is lost, leading to disruption of the BBB and eventually inducing severe brain injury [6]. These cells mentioned earlier are also involved in a variety of cell death processes, such as pyroptosis, apoptosis, necroptosis, iron apoptosis, and autophagy, as well as the crosstalk between these cell death modalities, which adds to the complexity of stroke pathogenesis [7]. Understanding the mechanism of death of these cells at the site of stroke prior to BBB destruction is important for the treatment of stroke.

Pyroptosis is one of the hottest topics in the current debate on cell death modalities, unlike apoptosis, which is a form of cell death associated with neuroinflammation [8]. The earliest studies on pyroptosis were related to tumors; however, in recent years, the field of pyroptotic cell death research has expanded to include many brain diseases [9]. Studies have recently demonstrated that pyroptosis mediates neuroinflammation after stroke and suggest pyroptosis-related pathways as potential therapeutic targets. Although the critical role of pyroptosis in stroke has been extensively researched, little is known about the cell types that undergo pyroptosis after stroke. Neuronal and glial cells are among the most frequently reported cells that undergo pyroptotic cell death after stroke; however, the intersection of pyroptosis and stroke appears to extend beyond these cells [10].

In this review we aim to summarize the latest mechanisms of action of pyroptosis, highlight the role of pyroptosis in the development of stroke, and list some potential therapeutic agents for stroke by targeting inhibition of the pyroptosis pathway. Overall, we provide this information that may provide some therapeutic strategies for the clinical development of new stroke drugs.

### Molecular mechanisms of pyroptosis

Pyroptosis was first observed in 1986 but was labeled “apoptosis” for almost the next decade, despite being found to regulate cell death along with inflammation and plasma membrane rupture, until 2001 when it was given its name “pyroptosis” [9]. Over the next two decades, insights have been gained into pyroptosis, which is a Caspase-dependent form of cell death.

### The “gasdermin” and “Caspase” families

The gasdermin family is considered a key executioner that regulates pyroptosis in various diseases. This family opens non-selective ion channels by non-specific binding to the cytoplasmic membrane, thereby contributing to cell swelling, ultimately leading to cell rupture and the release of large amounts of contents that promote inflammation [11]. Gasdermin (A/B/C/D/E) have all been shown to mediate the onset of pyroptosis, with gasdermin D (GSDMD) being the most closely associated with pyroptosis. The current mechanism of GSDMD-induced pyroptotic cell death is considered a general feature of pyroptosis. GSDMD possesses two conserved structural domains: an N-terminal (NT) functional domain and a C-terminal (CT) self-repressed structural domain, which are connected by a loop structural domain between the two ends [12]. GSDMD itself has not been reported to be cytotoxic and is unable to perform cell scavenging functions until it is modified, as the CT of GSDMD oligomerizes with NT and inhibits NT function. N-GSDMD acts by binding to lipids in the cell membrane to form pores, which occurs only on the inner side of the cell, and extracellular free N-GSDMD is unable to trigger pyroptosis [13]. Little is currently known about the function of C-GSDMD, except that it inhibits NT function. In addition, some studies have shown that the binding of Caspase-1 to GSDMD occurs mainly in CT, providing more ideas for the development of GSDMD-targeted inhibitors [14]. The Caspase family was thought for a long time to be associated only with apoptosis, and even though Black et al. [15] demonstrated in 1989 that Caspase-1 (then named “ICE”) activates pro-IL-1β processing and modification to mature IL-1β to induce cell death, it was not associated with pyroptosis at that time [16]. Further research revealed many differences between pyroptosis and apoptosis, and the “pyro” in pyroptosis reflects its relationship with inflammation. Unlike apoptosis, pyroptosis can be considered a pathological form of cell death. Pyroptosis-induced cell death is characterized by rupture of the plasma membrane, random loss of DNA fragments, and release of pro-inflammatory factors, among others [17]. A currently accepted pathway through which the Caspase family regulates the gasdermin family is that Caspase-1 induces pyroptosis by cleaving GSDMD. Not only does Caspase-1 cleave GSDMD into N-GSDMD (31 kDa) and C-GSDMD (22 kDa), but it is also involved in the activation of pro-IL-18 and pro-IL-1β into pro-inflammatory IL-18 and IL-1β, and the cleaved N-GSDMD binds to the cytoplasmic membrane to form pores to promote the release of inflammatory factors to mediate inflammation, which is also known as the classical inflammatory pathway [18]. Other members of the Caspase family, such as Caspases-4/5/11 and Caspases-3/8, are also thought to be involved in pyroptosis, although their roles are less significant [19–21].

### Characteristics of pyroptosis: spatio-temporal specificity

There is a temporal sequence between the rupture of the plasma membrane and the release of a large number of pro-inflammatory factors as a result of pyroptosis [22]. In addition, some inflammatory factors can be released before cell lysis and death, greatly contributing to inflammation [23]. Herein, we describe this process as an example of Caspase-1-induced pyroptosis. Host cells that receive foreign danger signals, such as pathogen-associated molecular patterns (PAMPs) and damage-associated molecular patterns (DAMPs), increase nuclear transcription of nuclear factor-*κ*-gene binding (NF-κB), which accelerates the synthesis of inflammasomes. Inflammasomes activate procaspase-1 into Caspase-1 with cleavage capacity, which subsequently cleaves GSDMD into functional NT and auto-inhibitory CT. N-GSDMD migrates to the host cell membrane to form a 10–20 nm pore, which does not rely on any N-GSDMD-specific receptors on the cell membrane [24]. The formation of GSDMD pores leads to the opening of ion channels that promote K^+^ efflux and Ca^2+^ influx, causing cell swelling and lytic death; it also mediates inflammation by allowing the release of the small-molecule protein IL-1 rather than pro-IL-1. However, it is important to note that the release of IL-1 occurs prior to the rupture of the plasma membrane and can be independent of pyroptosis. A recent study suggested that the GSDMD pore may discharge IL-1 via electrostatic forces, leaving the precursor (pro) that contains the acidic structural domain in the cell. This also explains why the precursor (pro), which is close in size to the mature IL-1 molecule, does not pass through the GSDMD pores [25]. In addition, the small inner diameter of the GSDMD pore prevents the release of some large pro-inflammatory factors, such as TNF-α, IL-6, and high mobility group protein 1 (HMGB1), which can only be detected after cell lysis and death. This characteristic of pyroptosis contributes to the transmission of neuroinflammation and accelerates the death of host cells [12].

### Inflammatory pyroptosis pathways

**Fig. 1:**
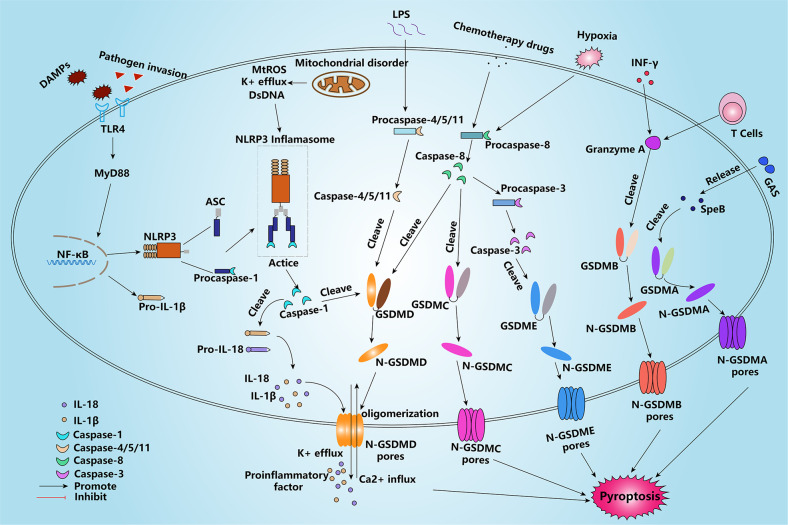
Mechanism of cell death induced by pyroptosis. In the classical inflammatory pathway, DAMPs and PAMPs increase the activation of NLRP3 inflammasomes, thereby promoting Caspase-1 cleavage of GSDMD and the pro-inflammatory factors IL-18 and IL-1β, causing pyroptosis; in the non-classical inflammatory pathway, LPS directly induces Caspase-4/5/11 to cleave GSDMD, thereby promoting pyroptosis. In addition to GSDMD, GSDMA/B/C/E can also induce pyroptosis and are not functionally significantly different in that they are cleaved by SpeB (secreted by GAS), Granzyme A, Caspase-8, and Caspase-3, respectively. DAMPs damage-associated molecular pattern molecules; TLR4 toll-like receptor 4, NF-κB nuclear factor kappa-B, NLRP3 NOD-like receptor thermal protein domain associated protein 3, ASC apoptosis-associated speck-like protein containing CARD, IL-18 interleukin-18, IL-1β interleukin-1β, dsDNA double-stranded DNA, LPS lipopolysaccharides, GSDMA/B/C/D/E gasdermin A/B/C/D/E, INF-γ interferon-γ, GAS group A Streptococcus, SpeB streptococcal pyrogenic exotoxin B.

Pyroptosis regulates cell death through two main pathways: a classical inflammatory pathway mediated by Caspase-1 and a non-classical inflammatory pathway mediated by Caspase-4/5/11 (Fig. 1). Caspase-1 can cleave GSDMD to N-GSDMD with pore-forming activity; however, it is generally present as an inactive precursor in normal living cells and is only cleaved to its active form after assembly into inflammasomes, which prevents Caspase-1 from excessively triggering inflammation [26]. Inflammasomes comprise three main components: a sensor, an adapter, an effector. Sensors of inflammasomes include the NOD-like receptor (NLR)-containing family (NLRP1, NLRP3, and NLRC4), absent in melanoma 2 (AIM2), and pyrin, which contain pattern recognition receptors (PRR) on their surface for recognizing information, and a caspase recruitment domain (CARD) or pyrin domain (PYD) [27]. The adapter for inflammasomes refers to the apoptosis-associated speck-like protein containing a CARD (ASC), which contains PYD and CARD structural domains at both ends. When the PRR on the surface of inflammasomes receives external danger signals, such as K^+^ efflux, ROS release, or double-stranded DNA (dsDNA) damage, it recruits large amounts of ASC via PYD-PYD. CARD-CARD oligomerization results in ASC with a sensor attached to one end and procaspase-1 containing a CARD structural domain attached to the other. The assembled inflammasome converts procaspase-1 into active Caspase-1 via self-cleavage [28]. Caspase-1 cleaves GSDMD into N-GSDMD, which binds directly to the plasma membrane and forms pores, leading to the opening of ion and molecular channels; this results in cellular hypertonicity and, ultimately, cell lysis and the release of large amounts of content to promote inflammation. As specific knockdown of Caspase-1 does not completely block pyroptosis, a non-classical pyroptosis pathway was discovered. Unlike Caspase-1, Caspase-4/5 (human-derived) and Caspase-11 (mouse-derived) also cleave GSDMD, leading to pyroptosis upon Lipopolysaccharide (LPS) stimulation, although they do not respond to the recruitment of inflammasomes and are unable to cleave pro-IL-1. Nevertheless, it is still thought that Caspase-4/5/11 is involved in the inflammatory pathway, given that regulating Caspase-1 expression by promoting K^+^ efflux promotes inflammation [29].

### Non-inflammatory pyroptosis pathways

Several Caspases involved in the inflammatory pyroptosis pathway have a cleavage effect only on GSDMD despite the presence of more than one executor of pyroptosis. In addition to the mainstream GSDMD-induced pyroptosis pathway, other pyroptotics are activated by other members of the gasdermin family. Caspase-3 cleavage of GSDME-induced pyroptosis, which is induced by treatment with chemotherapeutic agents, has received much attention in recent years [20]. Interestingly, Caspase-3 also plays a central role in the regulation of apoptosis. There is no evidence of a direct link between pyroptosis and apoptosis; however, it is certain that Caspase-3-mediated pyroptosis is inhibited during periods of high apoptotic expression [17]. As an upstream regulator of Caspase-3, Caspase-8 contributes to pyroptosis in many other ways besides regulating the cleavage of GSDME by Caspase-3 [30]. It was shown that catalytic Caspase-8 promotes the assembly of ASC-procaspase-1, in which Caspase-8 acts as a scaffolding protein [31]. Caspase-8 can cleave GSDMD to induce pyroptosis, similar to Caspase-1; under hypoxic conditions, nuclear transcription of GSDMC increases and Caspase-8 cleaves GSDMC into N-GSDMC to induce pyroptosis after TNF-α stimulation [32]. Furthermore, in tumor cells, granzyme A (GZMA) receives IFN-γ released from lymphocytes that cleave GSDMB to produce a similar result to GSDMD-induced pyroptotic cell death, thereby implementing positive feedback regulation to suppress tumors [33]. A recent report indicated that GSDMA can be cleaved into an NT active fragment by the group A Streptococcus (GAS) cysteine protease streptococcal pyrogenic exotoxin B (SpeB) virulence factor to trigger pyroptosis [34]. The activation of both GSDMA and GSDMB seems to be independent of the Caspase family, suggesting that they may be benign “killers”.

### The role of pyroptosis in stroke

**Fig. 2:**
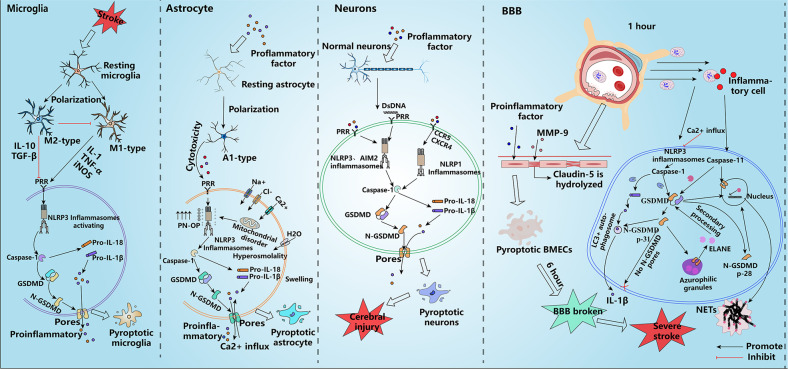
Types of pyroptotic cell death after stroke. After stroke, dormant microglia first rush to the damaged area and polarize into M1 and M2 types to exert pro-inflammatory and anti-inflammatory effects, respectively. However, under various stressful stimuli, microglia undergo pyroptosis and are converted to the M1 type, aggravating brain damage. Pro-inflammatory factors stimulate astrocytes to undergo pyroptosis, leading to increased membrane osmotic pressure and consequent water absorption and swelling, ultimately causing massive brain edema after stroke. Neuronal cells are more likely to undergo pyroptosis after losing the protection of astrocytes, resulting in irreversible brain damage. Pro-inflammatory substances released by microglia and astrocytes, among others, contribute to the pyroptosis of BMECs, accelerating the disruption of the BBB. In addition, GSDMD may exert other unknown effects in neutrophils; it seems to promote the release of NETs to induce secondary phagocytosis rather than directly triggering neutrophil rupture and death. IL-10 interleukin-10, TGF-β transforming growth factor-β, IL-18 interleukin-18, IL-1β interleukin-1β, GSDMD gasdermin D, dsDNA double-stranded DNA, TNF-α tumor necrosis factor-α, iNOS induced nitrogen monoxide synthase, PN-OP protein nanoparticle-induced osmotic pressure, AIM2 absent in melanoma 2, TLR4 toll-like receptor 4, CCR5 CC-chemokine receptor 5, CXCR4 chemokine (C-X-C motif) receptor 4, NLRP1 NOD-like receptor thermal protein domain associated protein 1, NLRP3 NOD-like receptor thermal protein domain associated protein 3, BBB blood-brain barrier, MMP-9 matrix metalloproteinase-9, BMECs brain microvascular endothelial cells, ELANE neutrophil elastase, NETs neutrophil extracellular traps.

A variety of cells are involved in the process of stroke, and both microglial activation and neutrophil infiltration are beneficial in early brain injury. However, continuous inflammation leads to massive brain tissue damage, eventually causing severe brain injury, and pyroptosis plays an important role in this process. Herein, we describe several cells that can undergo pyroptotic cell death during stroke, and pyroptosis uses these cells as a fuse to gradually “burn” the brain (Fig. 2).

### Microglia

Microglia are resident immune cells in the brain that rest under normal physiological conditions and have many protuberances that can be used to identify information, which enable them to reach the area of brain damage to play a neuroprotective role [35]. After stroke, microglia polarize from a dormant state into extreme M1 and M2 patterns. M1-type microglia release large amounts of pro-inflammatory factors such as IL-1, TNF-α, and iNOS to drive inflammation. In contrast, M2-type microglia can release the anti-inflammatory factors IL-10 and TGF-β to inhibit the transformation of the M1-type while also acting as phagocytes to clear cell debris [4]. In the early stage of stroke, the M2-type dominates, but after approximately one week, the positions are reversed, which is aided by pyroptosis. TLR4 on microglial membranes recognizes PAMPs and DAMPs and then activates the NF-κB pathway through its junction molecule MyD88 to increase the synthesis of NLRP3 and pro-IL-1β. NLRP3 rapidly assembles with ASC and procaspase-1 to form NLRP3 inflammasomes in response to stimuli such as K^+^ efflux, ROS release, and dsDNA damage, thereby activating the typical pyroptosis pathway. The formation of N-GSDMD pores not only promotes the early release of the pro-inflammatory factor IL-1 (IL-1 release is not entirely dependent on cell pyroptosis) but also accelerates microglial cell death. It is worth noting that M1 and M2 types are not just two types of cells; they have many subpopulations and dynamic patterns, and the balance between M1-type and M2-type cells determines the course of microglia-induced inflammation. The pre-stroke period is dominated by the M2 type; hence, initial neuroinflammation is beneficial to the patient, whereas the M1 type gradually dominates as the inflammation progresses [36]. Eventually, the balance between the M1 and M2 types is completely reversed approximately one week after stroke.

The bi-directional effects of microglia show that both inhibition of M1 expression and promotion of microglial conversion to the M2 type can help reduce stroke symptoms. Microglia are the first line of defense following stress injury or pathogen invasion after a stroke and are the earliest macrophages to mediate inflammation. It can be argued that the inflammatory response induced by pyroptotic microglia is a key cause of death in other cells [37]. Therefore, modulating the balance between M1 and M2 type patterns in microglia might help reduce post-stroke injury.

### Astrocyte

Both astrocytes and microglia are glial cells, and the presence of cerebral edema after stroke is closely associated with astrocyte swelling. Recent research has shown that pyroptosis is closely related to the swelling process in astrocyte [38]. The death of astrocytes by swelling due to pyroptosis can be broadly divided into two processes: the first process is the formation of surface membrane pores in a hyperosmotic state during which astrocytes release neurotoxins and lose their physiological function [39]. Upon sensing DAMPs or PAMPs, astrocytes are converted to the A1-type to release cytotoxins that damage neurons and induce intracellular inflammasome assembly to trigger pyroptosis, a small amount of which forms pores that can act as small molecule channels and non-selective ion channels, leading to Ca^2+^ influx [40]. Minimal influx of Ca^2+^ in the early stages may be beneficial for pyroptotic astrocytes as it maintains the ionic balance of astrocytes and can repair plasma membrane damage via chelation, which may inhibit pore formation to some extent [41]. As neuroinflammation progresses, a large Ca^2+^ influx causes endoplasmic reticulum stress (ERs), which results in Ca^2+^ being transported into the mitochondria, causing mitochondrial Ca^2+^ overload and, consequently, mitochondrial dysfunction [42]. Mitochondrial dysfunction promotes intracellular inflammasome assembly and further amplification of Ca^2+^ influx, thereby increasing protein nanoparticle-induced osmotic pressure (PN-OP) on astrocyte membranes, leading to astrocyte swelling. Zheng et al. showed that inflammasomes acting as PN-induced OP are required for astrocyte plasma membrane swelling and that astrocyte swelling is significantly reduced following the inhibition of ASC and Caspase-1 expression, which are important components of inflammasomes [38]. During the second phase of astrocyte pyroptosis, astrocytes lose their physiological functions and are reduced to amplifying agents of inflammation. The influx of large amounts of ions causes astrocytes to become hyperosmotic, inducing the opening of aqueous ion channels, causing astrocytes to swell continuously (irreversible swelling) and eventually rupture and die [43]. Astrocyte rupture results in a massive outflow of content, further promoting inflammation. Similar to microglia, astrocytes, which undergo pyroptosis after stroke, also mediate neuroinflammation.

Astrocytes are important for the regulation of brain homeostasis. They act as housekeepers of brain neurons and are indispensable for normal neuronal activity. Astrocytes have abundant protrusions that not only separate neurons but are also responsible for transporting nutrients to neurons from blood vessels and acting as ionic buffer stations to regulate the altered ionic environment that results from neuronal cell activity. Neurons lose a comfortable environment that they depend on for survival after astrocytes undergo pyroptotic cell death, resulting in disruption of neuronal cell metabolism and increased brain damage [44]. In addition, astrocytes, together with BMECs and peripheral cells, constitute the BBB and are important for maintaining its integrity [45]. Due to the loss of astrocyte support, the BBB is destroyed just hours after stroke, leading to an influx of substances harmful to brain cells, exacerbating the inflammatory response.

### Neuron

Neurons are important for normal life activities, and damage to neurons has serious consequences. Neuronal death is one of the end results of stroke and can lead to limited motor function, memory impairment, and even death. Much of the brain damage caused by stroke is related to neuronal damage; the higher the degree of neuronal damage, the greater the disability caused by stroke [46]. More than a dozen forms of neuronal cell death have been reported, of which pyroptosis has recently been proposed [7, 47]. We now know that neuronal pyroptosis is initially induced mainly by inflammatory factors released from glial cells and that inflammatory factors can induce the assembly of NLRP3 inflammasomes in neuronal cells, leading to neuronal death. Moreover, activated M1-type microglia also mediate the production and release of large amounts of ROS through HV1 channels, which have been shown to induce the assembly of NLRP3 inflammasomes, inducing neuronal pyroptosis [48]. AIM2 is an inflammasome that is highly expressed in neurons after stroke and, unlike the NLRP3 inflammasome, is only activated by cytoplasmic dsDNA [49]. AIM2 consists mainly of the N-terminal HIN structural domain and the C-terminal PYD structural domain, which recognizes dsDNA and binds to ASC via the PYD structural domain to form inflammasomes. Many cells die after brain injury, resulting in abundance of free abnormal dsDNA in the blood. AIM2 in neuronal cells detects these dsDNA and rapidly assembles AIM2 inflammasomes, and it has been shown that AIM2 also promotes Caspase-1 activation and is involved in the cleavage of IL-1β, IL-18, and GSDMD. Although AIM2 is essential for normal human brain development, it also exacerbates the inflammatory response during stroke [50]. In addition to NLRP3 and AIM2 inflammasomes, NLRP1 inflammasomes have been shown to be expressed primarily in neuronal cells and to cleave GSDMD as well as activate IL-1β. NLRP1 is predominantly expressed in neuronal cells after stroke and, owing to its unique pyrin structural domain, can bind directly to procaspase-1, thereby facilitating Caspase-1 cleavage of GSDMD with the pro-inflammatory factor IL-1 [51]. The chemokine receptors CCR5 and CXCR4 on neuronal cells have all been shown to be involved in the assembly of NLRP1 after stroke [52, 53]. The pyroptotic neuron pathway appears to be highly dependent on the Caspase-1 pathway; thus, targeting Caspase-1 inhibition can effectively reduce neuronal pyroptosis. TAK1 (TGF-β activated kinase 1) has also been reported to induce NLRP3 assembly during stroke by activating NF-κB, increasing ROS release, and promoting lysosomal autophagy. Notably, targeted inhibition of TAK1 appears to promote Caspase-8-mediated pyroptotic cell death in neuronal cells [54, 55].

Neuronal cells are not the ultimate target of pyroptosis as they also partially mediate inflammation and transmit these pro-inflammatory factors through the bloodstream to induce pyroptotic cell death, thereby exacerbating brain damage. This means that in the ischemic penumbra, as blood flows, any type of cell involved in pyroptotic cell death can contribute. In addition, damaged neurons are difficult to repair after a stroke; therefore, the issue of neuronal safety is key to addressing post-stroke brain injury.

### BMECs

BMECs are among the main components of the BBB and are tightly bound to other cells (astrocytes, pericytes, etc.) through tight junction and adhesion junction proteins, which together form a barrier that controls the entry and exit of substances harmful to the brain [56]. The degree of BBB damage is closely associated with the degree of stroke deterioration. As the integrity of the BBB is disrupted, harmful substances in the blood circulation can further induce brain tissue damage, thereby exacerbating stroke [57]. We always focus on neuronal cells and glial cells after a stroke and often overlook the role of pyroptotic BMECs in the early stages of stroke. BMECs can also undergo pyroptosis, which has rarely been reported [58]. Approximately one hour after stroke, the BBB permeability increases, allowing neutrophils to pass through the BBB to the site of inflammation in the brain, which is closely related to the structural changes in BMECs. After stroke, the structure of BMECs is altered as glial cells release MMP-2 and MMP-9 to hydrolyze tight junction proteins (such as claudin-5 and occludin) on the cells [59]. At the same time, BMECs undergo internal pyroptosis and may be degraded simultaneously or earlier than the external ligand proteins of BMECs, the interaction of which results in fragile BBB integrity after stroke [6]. Early in the stroke, pyroptotic BMECs may be induced primarily by IL-1 and ROS released from microglia, such that the result is a rearrangement of the carbon skeleton and loss of ligand protein function in the BMECs, ultimately causing initial BBB damage. BMECs contain a large number of mitochondria; therefore, after pyroptosis, a large amount of ROS is released due to mitochondrial dysfunction, and these ROS induce damage to other cells that make up the BBB, thereby disrupting the integrity of the BBB [60, 61]. Moreover, BMECs are inflammatory cells that undergo pyroptotic cell death and release a large number of inflammatory factors, particularly intercellular cell adhesion molecule-1 (ICAM-1), which attracts the attention of neutrophils. By transiently binding to ICAM-1 on BMECs, neutrophils lend themselves by migrating from the capillary wall to the site of inflammation in the brain, also known as neutrophil infiltration [62].

The integrity of the BBB is the last line of defense to ensure that substantial damage to the brain occurs after stroke. As pyroptotic BMECs develop mainly in the early stages of stroke, the direct damage to the brain cannot be fully explained; however, the destruction of the BBB indicates that the brain enters an irreversible cycle of inflammation. Pyroptotic BMECs largely contribute to the impairment of BBB integrity, which, in turn, facilitates the entry of various inflammatory factors into the ischemic penumbra to accelerate pyroptotic cell death. Therefore, we believe that ameliorating pyroptotic BMECs during early stroke may be a key target to prevent stroke progression.

### Neutrophil

Neutrophils are the most abundant class of immune cells in the body and are highly expressed at the sites of inflammation induced by various bacterial infectious diseases. Neutrophils have a short life cycle and are typically found in capillaries [63]. However, once they sense an invasion of pathogens or inflammatory cellular response, they migrate to the site of inflammation to engulf these cells and protect the body under the action of chemotactic substances. In particular, the onset of neuroinflammation during stroke attracts a large number of neutrophils to migrate from capillaries to the site of inflammation. Thus, neutrophil infiltration is considered a typical feature of stroke [64]. Neutrophils also undergo pyroptosis after a stroke; however, unlike macrophages, neutrophils are resistant to Caspase-1-induced pyroptosis. Although neutrophils have functional NLRP3 and NLRC4 inflammasomes that recruit Caspase-1, Caspase-1 is not essential for neutrophils, and the inflammatory function of neutrophils is largely dependent on Caspase-11. In fact, pathogen invasion and neuroinflammation cause the assembly of inflammasomes within neutrophils after a stroke, and self-cleaving Caspase-1 also mediates the cleavage of GSDMD and the maturation of IL-1β; however, the number of pores sufficient for pyroptosis of neutrophils could not be observed [5]. Other studies have shown that although the inflammatory response is promoted by “pyroptotic neutrophils”, the associated pro-inflammatory factors, such as IL-1β, appear to be released via the autophagic pathway rather than GSDMD pores [65].

Intriguingly, although neutrophils can release IL-1β independently of the pores of pyroptotic cells, they are largely inseparable from the role of GSDMD. Karmakar M et al. found that N-GSDMD cleaved by Caspase-1 in neutrophils could localize to asplenophilic granules and LC3^+^ autophagosomes, and they made the reasonable conjecture that N-GSDMD binds more strongly to organelle membranes than to plasma membranes within neutrophils, which may be related to the powerful membrane repair mechanism of the neutrophils [65]. N-GSDMD promotes the transport of mature IL-1β to LC3^+^ vesicles, thereby releasing IL-1β and maintaining the inflammatory effects of neutrophils. Moreover, N-GSDMD forms small pores in asplenophilic granules, allowing the release of neutrophil elastase (ELANE) into the cytoplasm, which can secondarily process GSDMD into 28 kD N-GSDMD (functionally not significantly different from 31 kD N-GSDMD).

The plasma membrane of neutrophils does not appear to be completely lysed after pyroptotic cell death, as is the case with macrophages, but forms a structure of neutrophil extracellular traps (NETs) that can exert a secondary anti-inflammatory effect [66]. Caspase-11, another member of the Caspase family in neutrophils, is also thought to be significantly associated with neutrophil death. Interestingly, Caspase-11-cleaved GSDMD can localize to the nuclear membrane and cause the appearance of pores in the nuclear membrane, allowing Caspase-11 to enter the nucleus. The Caspase-11-mediated expansion of DNA to reach the conditions for NET formation provides the first indication of a way of death other than gasdermin family-mediated pyroptosis [67]. Despite the involvement of the gasdermin family, Caspase-11-mediated NETs differ from what we perceive as pyroptosis in that there is no complete plasma membrane rupture. This peculiar phenomenon is most likely due to the inhibition of Caspase-1 expression by neutrophils and the absence of Ca^2+^ influx [41]. In any case, we need to change our perception of the gasdermin family: although the cleaved NT of the gasdermin family binds to the plasma membrane to induce pyroptotic cell death in most monocytes, in some cells, such as neutrophils, it may preferentially select various organelle membranes (including the nuclear membrane) because the plasma membrane is not easily pore-forming.

### Crosstalk between pyroptosis, apoptosis, and necroptosis after stroke

Pyroptosis, apoptosis, and necroptosis are all genetically regulated forms of programmed cell death (PCD), which are essentially a way for the body to “clean up” in response to pathogen invasion or stress-induced tissue damage. Recent research supports the existence of crosstalk between these three forms of PCD (they can substitute or transform each other) [68]. It is worth mentioning that pyroptosis occurs mainly in the infarct border zone, while apoptosis occurs more often in the infarct core zone. Even though we already know that Caspase-8 may be a central target that regulates their crosstalk, we still do not know how these three act individually after stroke. Generally, when the death receptor (DC) or TLR4 on the host cell surface senses an exogenous stimulus signal (e.g., the death ligand TNF-α), it will likely undergo any of these forms of PCD, depending on the enzymatic activity of Caspase-8. When Caspase-8-related proteases are normally expressed, cells mainly undergo apoptosis. In contrast, when the enzymatic activity of Caspase-8 is inhibited by microbial secretions, the phosphorylated expression of RIPK1/RIPK3 increases, promoting MLKL phosphorylation and, eventually, the necrosome formed by MLKL oligomerization causes nanoscale pores in the cell membrane to induce necroptosis. Interestingly, inactive Caspase-8 was reported to act as a backbone protein to promote ASC-procaspase-1 linkage and pyroptosis. Caspase-8 can also directly cleave GSDMD to induce pyroptosis when TAK1 is inhibited [17, 47, 69]. This suggests that the three PCDs are interchangeable under specific conditions; however, this also obscures the specific roles of each PCD after stroke.

When PANoptosis (pyroptosis, apoptosis, and necroptosis) was proposed, it was found that these three types of PCD could act collectively to regulate cell death. PANoptosis is a more complex form of cell death crosstalk and its activation is highly dependent on the Z-DNA binding protein 1 (ZBP1)-RIPK3-PANoptosome. Upon receiving the signal of influenza A virus (IAV) intrusion, ZBP1-RIPK3 acts as a sensor to recruit corresponding adapters (e.g., NLRP3, ASC, and Caspase-8) to attract “hitters” (Caspase-1, Caspase-3/7, MLKL) and induce PANoptosis [70, 71]. Numerous experimental results indicate that PANoptosis can be triggered after stroke. On this basis, an increasing number of common targets for PANoptosis are being discovered. Caspase-6, previously thought to have no specific role other than the regulation of apoptosis, has recently been shown to promote the assembly of ZBP1-RIPK3-PANoptosome, thereby participating in the regulation of PANoptosis [72]. TAK1 is another protein that has recently received attention for its involvement in the regulation of PANoptosis. TAK1 has been previously reported to regulate the transition between apoptosis and necroptosis, and TAK1 inhibition promotes Caspase-8 cleavage in GSDMD-induced pyroptosis. In an I/R stroke model, inhibition of TAK1 triggered all three PCDs simultaneously and exacerbated the inflammatory response, suggesting that TAK1 is a key target for inducing cell death during cerebral ischemia/reperfusion injury (CI/RI) [73].

**Fig. 3:**
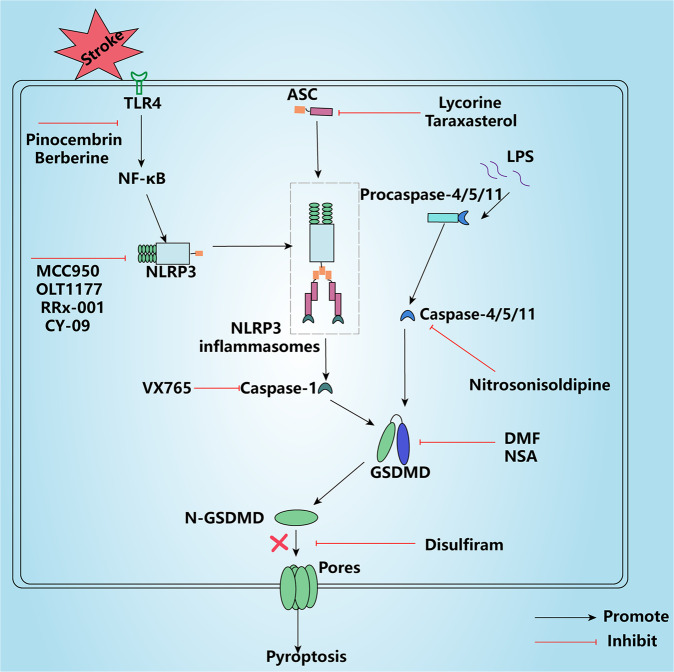
Crosstalk associations between the three types of PCD after stroke. After stroke, TLR4/DR on the cell membrane receives the corresponding inflammatory stimulus and triggers pyroptosis, apoptosis, or necroptosis. Caspase-8 is a key protein regulating several modes of PCD death; normal expression of Caspase-8 enzyme activity favors the induction of apoptosis, diminished Caspase-8 activity helps induce necroptosis, whereas inactive Caspase-8 can promote ASC-procaspase-1 binding to induce pyroptotic cell death. The PANoptosome, a key complex triggering PAN-optosis, is mainly activated by IAV invasion, in which ZBP1 and RIPK3 act as sensors and Caspase-6 enhances their sensitivity; the reduction of TAK1 after stroke also promotes PANoptosome formation, triggering PANoptosis and aggravating brain injury. TLR4 toll-like receptor 4, DR death receptor, RIPK1 receptor interacting serine threonine kinase 1, RIPK3 receptor interacting serine threonine kinase 3, MLKL mixed-lineage kinase domain-like protein, ZBP1 Z-DNA binding protein 1, NLRP3 NOD-like receptor thermal protein domain associated protein 3, ASC apoptosis-associated speck-like protein containing CARD, NF-κB nuclear factor kappa-B, GSDMD gasdermin D, IAV influenza A virus, PANoptosis pyroptosis + apoptosis+necroptosis, TAK1 TGF beta-activated kinase 1.

PANoptosis proposal provides many new targets for the treatment of stroke besides the existing targets. Targeted inhibition of PANoptosis can reduce neurological damage after stroke, allowing for better prognosis and recovery. However, whether PANoptosis occurs after a stroke may still depend on the different periods of the stroke and the type experienced by the patient. PANoptosis is triggered primarily by viral invasion, and although all three modes of death have been shown to occur simultaneously in various experimental stroke models (MCAO, OGD, I/R, etc.), there are few clinical data on whether PANoptosis can be triggered in every stroke patient [72]. In any case, PANoptosis represents a new way of regulating cell death in stroke (Fig. 3).

### Potential drugs for stroke—targeting pyroptosis pathway

There are three main classes of drugs currently used clinically for stroke treatment: thrombolytic drugs (rt-PAs, antithrombotic and antiplatelet aggregation), anti-atherosclerotic drugs (statins), and neuroprotective drugs. Neuroprotective drugs have not been clinically effective, but they have an important role in stroke prevention and recurrence. Pyroptosis plays an important role in the early stages of stroke, accelerating the destruction of the BBB and leading to severe brain damage. Therefore, targeted inhibition of the pyroptosis pathway is a promising strategy to reduce stroke injuries.

### Stroke agents that inhibit the activation of NLRP3 inflammasome

Numerous experiments have shown that inhibition of the upstream pathway of pyroptosis can significantly improve stroke symptoms [74]. In recent years the NLRP3 inflammasome has been identified as a potential therapeutic target for the treatment of immune diseases, it is involved in a wide range of inflammatory activities, not only that, it is also a key player in the regulation of pyroptosis, however so far no drugs have been developed clinically to target the NLRP3 inflammasome to treat disease. Two signals are required for the NLRP3 inflammasome to perform its function: the NF-κB signaling pathway, which is responsible for the synthesis of NLRP3 and the associated inflammatory molecule pro-IL-1β; and endogenous stress stimuli, such as mtROS, dsDNA, and K^+^ efflux, which promote the recruitment of ASC and procaspase-1 by NLRP3 for assembly into inflammasomes [75]. The natural product pinocembrin possesses pleiotropic neuroprotective effects and is currently in a phase II clinical trial for ischemic stroke. Pinocembrin exerts anti-inflammatory effects by inhibiting the activation of the TLR4/NF-κB pathway and the activation of m1 phenotype microglia, in addition to reducing the hemorrhagic transformation that results from t-PA treatment [76, 77]. Berberine, a compound extracted from Coptis chinensis, has good antibacterial and anti-inflammatory properties. Berberine exerts anti-inflammatory effects by inhibiting the TLR4/NF-κB pathway, in addition to its anti-platelet aggregation and anti-atherosclerosis properties, making it a promising drug for the treatment of stroke [78]. MCC950, a specific inhibitor of the NLRP3 inflammasome, inhibits NLRP3 activation mainly by interacting with the Walker B motif in the NACHT structural domain of NLRP3 and by affecting the ATP hydrolysis of NLRP3, thereby reducing cell death, and may be considered a candidate for stroke treatment. In addition, prophylactic administration of MCC950 reduced the rate of cerebral infarction by ~60% in mice with cerebral ischemia [79, 80]. Dapansutrile (OLT1177) is an oral specific NLRP3 inhibitor that is harmless to humans. Its exact mechanism of action on NLRP3 is unknown; however, its ability to effectively block NLRP3 activation and reduce the synthesis of the pro-inflammatory factor IL-1β has allowed its use for painful strokes and makes it a promising treatment option for stroke [81]. RRx-001 is a promising selective inhibitor of NLRP3 that mainly acts by binding to cysteine 409 of NLRP3 to block the NLRP3-NEK7 interaction, which is extremely important for NLRP3 activation. RRx-001 is currently in phase III clinical trials and is a relatively safe drug that has the potential to become a treatment for stroke [82]. CY-09 inhibits NLRP3 activation by binding to the Walker A motif of NLRP3 and reducing NLRP3 binding to ATP; similar to MCC950, it inhibits NLRP3-dependent inflammatory diseases and could be a potential drug for the treatment of stroke [83].

Activation of NLRP3 inflammasomes is highly dependent on ASC recruitment of free procaspase-1 and NLRP3 activation; therefore, targeted inhibition of ASC could also be a therapeutic strategy for stroke. Lycorine (LYC) is an anti-inflammatory agent that is extracted from the Lithospermum family and inhibits the expression of active Caspase-1 and related molecules of the downstream pyroptosis pathway by targeting the PYD structural domain of ASC to reduce the activation of NLRP3 inflammasomes, thereby reducing pyroptosis [84]. Similarly, taraxasterol (TAS), an anti-inflammatory agent extracted from Taraxaster, inhibits the assembly of NLRP3 inflammasomes and pyroptosis by reducing ASC spot formation. Not only that, TAS also reduces neuronal damage by countering oxidative stress and apoptosis through the Nrf2 antioxidant pathway [85]. It is well known that pyroptosis is highly dependent on Caspase-1; thus, Caspase-1 inhibitors also have great potential as drugs for stroke treatment. VX765 is a Caspase-1 inhibitor that not only inhibits the downstream pyroptotic pathway of Caspase-1 (including reducing the expression of the pro-inflammatory factor IL-1 and the pore-forming protein N-GSDMD), but also inhibits ASC oligomerization, which helps to reduce neurological damage after stroke. Like MCC950, VX765 is one of the most commonly used inhibitors in stroke models, so it may have a better effect for stroke treatment [86]. Nitrosonisoldipine is a calcium channel blocker, mainly used for the treatment of cardiovascular system diseases. Also nitrosonisoldipine is an inhibitor of the Caspase family, which possesses good inhibitory effects on both Caspase-1 and Caspase-11 and can inhibit pyroptosis, and therefore can be a candidate for stroke treatment [87].

### Stroke agents that inhibit GSDMD activity

**Fig. 4:**
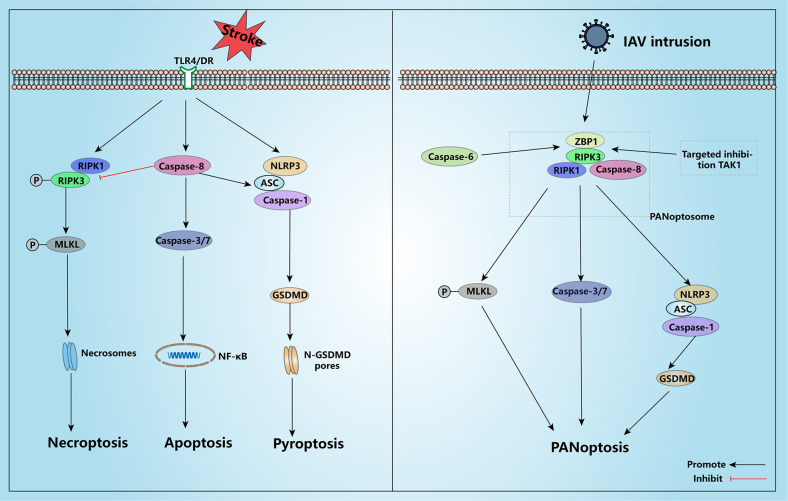
Potential drugs for stroke—targeting pyroptosis. Pinocembrin and berberine ameliorate neuroinflammation and pyroptosis after stroke by inhibiting the NF-κB pathway activation, thereby reducing the activation of NLRP3 inflammasomes. MCC950, OLT1177, RRx-001, and CY-09 reduce post-stroke pyroptosis by inhibiting NLRP3 inflammasome activation, whereas lycorine and taraxasterol act by affecting the structural domain of ASC, indirectly blocking NLRP3 inflammasome activation. VX765 and nitrosonisoldipine improve post-stroke neuroinflammation and pyroptosis by inhibiting Caspase-1 and Caspase-4/5/11, respectively. DMF and NSA reduce pyroptotic cell death by targeting Caspase-1-mediated processing of GSDMD at Cys191/Cys192. Disulfiram reduces pyroptosis by directly affecting the pore-forming activity of N-GSDMD. JMZ trimetazidine, OLT007 dapansutrile, NSA necrosulfonamide, DMF dimethyl fumarate, LPS lipopolysaccharides, NLRP3 NOD-like receptor thermal protein domain associated protein 3, ASC apoptosis-associated speck-like protein containing CARD, GSDMD gasdermin D.

GSDMD is the last downstream substrate of the pyroptosis pathway and has the closest relationship with pyroptotic cell death; therefore, targeted inhibition of GSDMD may be a viable option for the treatment of stroke (Fig. 4) [88]. While Disulfiram has been used as a drug for alcohol addiction in the past, Hu JJ et al. reassessed its action and defined it as a direct inhibitor of GSDMD. Disulfiram inhibits pore formation mainly by modifying the Cys^191^/Cys^192^ of GSDMD. Although this process does not seem to affect the modification of GSDMD and IL-1 by Caspase-1, the disappearance of GSDMD pores largely reduces the occurrence of pyroptotic cell death [89, 90]. Compared to most Cys-modified drugs, disulfiram exhibited strong activity against GSDMD with no clinically significant toxicity, making it a promising inhibitor of pyroptosis. Mechanistically, necrosulfonamide (NSA), an inhibitor of necroptosis, can also inhibit the oligomerization of GSDMD dimers, preventing the formation of GSDMD pores without affecting the upstream pathway. NSA also acts by targeting the direct binding of Cys^191^ to GSDMD, and although this targeting specificity may be affected by other cysteine-containing molecules, it remains a promising inhibitor of pyroptosis [91]. The fate of the processed GSDMD after it fails to bind to the cytoplasmic membrane and whether it performs other unknown functions are worth investigating; for instance, a previous report suggested that GSDMD can trigger the release of NETs. Dimethyl fumarate (DMF) is a preservative with powerful antimicrobial properties that is classified as non-edible due to its corrosive properties in humans; however, it is clinically approved by the FDA for the treatment of multiple sclerosis (MS). In recent studies, DMF was found to block the processing of GSDMD by Caspase-1, inhibit GSDMD oligomerization, and reduce cell death. GSDMD is succinated by DMF at Cys^191^/Cys^192^, resulting in loss of protein function and, consequently, an inability to be processed by Caspase-1 and a loss of oligomerization pore-forming ability. Other fumarate analogs also exhibit some anti-pyroptosis effects. In addition, GSDME was found to be succinated by DMF in GSDMD-deficient cells [88, 92]. Conceivably, these drugs have a preference for the Cys^191^ (in humans)/Cys^192^ (in rats) of GSDMD, suggesting that we can focus on drugs that can bind to these residues when screening for pyroptosis inhibitors.

### Stroke agents that regulate the M1/M2 polarization of microglia

Microglia are known to mediate the post-stroke inflammatory cascade, and M1/M2 microglia play opposing roles after stroke. Inhibition of M1-type polarization or promotion of M2-type polarization could help reduce neuroinflammation and improve stroke symptoms. Resveratrol is a natural polyphenol that is often used as an anti-inflammatory and anti-oxidant drug and has neuroprotective effects. Studies have shown that resveratrol can inhibit M1 microglial polarization in an inflammatory environment and increase M2 microglial polarization via PGC-1α (peroxisome proliferator activated receptor coactivator-1α). Moreover, resveratrol pretreatment also prolonged the tolerance time window in cerebral ischemic mice by increasing glycolysis and mitochondrial respiratory efficiency [93, 94]. Telmisartan has been reported to reduce LPS-induced inflammatory responses in BV2 cells and mouse primary microglia, and to promote M2-type microglia polarization and inhibit M1-type polarization via AMPK/PGC-1α, suggesting that it could be developed as a stroke agent [95]. miR-155 is a micro-RNA (miRNA) involved in the biological processes of various inflammatory diseases and is also important for the regulation of M1/M2 microglia. Astragalus polysaccharide can enhance the immunity of human body, enhance the proliferation and differentiation of immune cells, and also dilate blood vessels, which is also useful for cardiovascular diseases. Studies have shown that astragalus polysaccharide regulates the polarization of M1/M2 microglia by inhibiting miR-155 expression, thereby reducing neuroinflammation [96]. Metabolic reprogramming is one of the key factors in the conversion of microglia to M2 in an inflammatory environment, and rosmarinic acid inhibits neuroinflammation by modulating the metabolic reprogramming of microglia and could be a potential therapeutic option [97].

### Stroke agents that regulate A1/A2 polarization of astrocytes

Although neuronal damage is the main cause of brain injury after stroke, pharmacological treatments used to protect neurons have failed to achieve good clinical outcomes, suggesting the need to consider the protection of multiple cell types. Astrocytes play an important role in the central nervous system and are biologically important for maintaining the BBB integrity and normal neuronal activity. In ischemic stroke, astrocytes are first polarized to the A1 phenotype (pro-inflammatory) by the activation of inflammatory factors released from M1 microglia. A1 phenotype astrocytes release inflammatory factors and neurotoxins that severely damage neurons, whereas the A2 phenotype (anti-inflammatory) plays a strong neuroprotective role. As a result, modulation of the A1/A2 phenotype astrocyte expression could protect neurons and reduce stroke symptoms. Cottonseed oil is often used as a solvent for lipid-soluble substances and therefore can cross the blood-brain barrier well. Recently, it was reported that cottonseed oil can reduce cerebral ischemic injury by inhibiting the expression of A1 phenotype astrocytes in rats with cerebral ischemia, which is an ideal drug for stroke [98]. Accumulation of ROS and mitochondrial dysfunction are also among the factors that induce the polarization of astrocytes. n-3 polyunsaturated fatty acids (n-3 PUFAs) inhibit A1 phenotypic polarization by reducing mitochondrial dysfunction, which contributes to post-stroke recovery [99]. Furthermore, Ligusticum chuanxiong Hort (LCH) can induce conversion of reactive astrocytes from the A1 to A2 phenotype [100].

In fact, stroke is a very complex pathological process, not only neuroinflammation, but also oxidative stress plays an important role. Natural medicines with anti-inflammatory and anti-oxidant effects can alleviate stroke symptoms to some extent. For example, ginkgo biloba extract is widely used in the clinical treatment of ischemic stroke. Ginkgo biloba extract, for example, is widely used clinically in the treatment of ischemic stroke and possesses good anti-oxidative stress and anti-neurotoxic effects, although it is more useful in improving vascular microcirculation [101]. Here we list some of the Traditional Chinese Medicines (TCMs) that have the potential to treat stroke (Table 1).

**Table 1:** TCMs to improve neuroinflammation and pyroptosis after stroke.

TCM drugs	Sources	Effects	Target points	Models	Refs.
Chrysophanol	*Rheum Palmatum* L.	Inhibits M1-type microglia activation and TRAF6-dependent pyroptosis	TRAF6/NLRP3	MCAO; OGD/R	[101, 106]
Isoquercetin	*Styphnolobium japonicum* (L.) Schott	Anti-inflammatory, antioxidant, and reduces neuronal apoptosis	TLR4/NLRP3; Caspase-3; Nrf2	MCAO/R; OGD/R	[107, 108]
Baicalin	*Scutellaria baicalensis* Georgi	Protects glutamine synthetase, antioxidant, and anti-inflammatory	AMPK/NLRP3; ROS	MCAO/R; OGD/R	[109, 110]
Astragaloside IV	*Astragalus membranaceus* (Fisch.) Bunge	Inhibition of pyroptosis and regulation of autophagy	Nrf2/NLRP3; GSDMD; LC3	MCAO/R; OGD/R	[111, 112]
Resveratrol	*Polygonum cuspidatum* Sieb. et Zucc.	Regulation of M1/M2 microglia, regulation of autophagy, and anti-apoptosis	PGC-1α; Sirt1; JAK2/STAT3/PI3K/AKT/mTOR	MCRO/R; OGD	[93, 113, 114]
Mangiferin	*Anemarrhena asphodeloides* Bge.	Anti-inflammatory, antioxidant, and anti-apoptotic	TLR4/NLRP3; SIRT1/PGC-1α	MCAO/R; OGD/R	[115, 116]
Salidroside	*Rhodiola rosea* L.	Anti-inflammatory, antioxidant, and anti-apoptotic	TLR4/NLRP3; Bax/Bcl-2; Nrf2/Trx1	MCAO/R; OGD/R	[117, 118]
Schisandrin B	*Schisandra chinensis* (Turcz.) Baill.	Inhibits neuroinflammation and reduces pyroptosis	NF-κB/NLRP3;	MCAO/R	[119, 120]
Berberine	*Coptis chinensis* Franch.	Inhibits neuroinflammation and reduces pyroptosis	PPAR-γ/NLRP3; GSDMD	OGD	[121]
Astilbin	*Astilbe chinensis* (Maxim.) Franch. et Savat.	Inhibits neuroinflammation and reduces apoptosis	TLR4/NLRP3; Caspase-3	MCAO; OGD	[122]
Medioresinol	*Eucommia ulmoides* Oliv.	Improves BBB integrity and inhibits pyroptosis	PGC-1α/PPARα; GSDMD	MCAO; OGD/R	[58]
Ginkgo diterpene lactones	*Ginkgo bilob*a L.	Inhibits astrocyte activation, anti-inflammatory, and antioxidant	TLR4/NF-κB; AKT/Nrf2	MCAO/R; OGD/R	[123, 124]
6-Gingerol	*Zingiber officinal*e Rosc.	Inhibits neuroinflammation and apoptosis and upregulates autophagy	TLR4/NLRP3;TRPV1/FAF1; Akt-mTOR-STAT3	MCAO/R; OGD/R	[125, 126]
Ruscogenin	*Ophiopogon japonicus* (L.f.) Ker-Gawl.	Inhibits neuroinflammation and improves BBB integrity	ROS-NLRP3; TXNIP; MAPK; ICAM-1	MCAO/R; OGD/R	[127, 128]
Dendrobium alkaloids	*Dendrobium officinale* Kimura et Migo	Inhibits pyroptosis	Caspase-1; GSDMD	OGD/R	[129, 130]

### Conclusion and perspective

Stroke imposes a huge economic burden worldwide, and the number of stroke victims increase each year [102]. There is no exact cause of stroke; it is usually caused by chronic diseases and occurs mostly in immunocompromised elderly people Strokes usually result from blockage of arteries in the brain. The onset of stroke is very rapid, with massive brain edema occurring within 24 h of onset, seriously endangering the patient’s life [103]. The stroke site is divided into the ischemic core and penumbra, where the penumbra is more active owing to the greater blood flow in the ischemic penumbra, which facilitates intercellular information transfer. Within 1–6 h after stroke, swelling and death of neuronal cells, astrocytes, BMECs, and other cells can be observed in the ischemic penumbra, followed by a large dispersal of inflammatory factors due to BBB destruction caused by massive endothelial cell death, which eventually leads to massive brain edema. Therefore, treatment within 6 h after a stroke can minimize brain damage, which requires a more in-depth understanding of this period [104]. Multiple modes of cell death are involved in the early stages of stroke, and pyroptosis plays an important role because it is not only responsible for programmed cell death, but also promotes neuroinflammation.

Pyroptosis is mainly caused by Caspase-1, which cleaves GSDMD of the gasdermin family into NT with pore-forming activity to bind to and form pores in the plasma membrane. Unlike the other two modes of programmed death, cells undergoing pyroptosis can release the small-molecule pro-inflammatory factors IL-18 and IL-1β to promote inflammation prior to membrane rupture [22]. After stroke, the injury induces dormant microglia to polarize into M1 and M2 types and simultaneously turns on a “switch” that induces pyroptotic microglia. Microglia deliver pro-inflammatory factors to neuronal cells and astrocytes via blood transmission, thereby inducing them to undergo pyroptosis. The end-foot of astrocytes covers 85% of the BBB, and as astrocytes die, ~1 h after stroke, endothelial cells begin to undergo pyroptosis and lose connexin function, which disrupts the BBB integrity [6, 57]. Simultaneously, inflamed cells at the site of stroke injury also attract the attention of neutrophils, which eliminate these cells by permeating the BBB. What is certain is that pyroptosis is a highly important driver in the early stages of stroke, and its suppression can delay the time to stroke progression and provide a larger treatment window.

All targeted neuroprotective drugs have failed to achieve satisfactory results in clinical trials for stroke treatment. The reason for this is that the onset of stroke is rapid and complex, and it is often difficult to achieve a therapeutic effect if we target only one stage of stroke in a one-sided manner [105]. However, neuroprotection remains an essential factor in the prognosis of stroke patients. In fact, the commonly used clinical treatments for stroke (mainly ischemic stroke), such as thrombolytic drugs or thrombectomy, do not guarantee an after-healing effect in stroke patients. Stroke-induced neuronal cell death is difficult to recover, even after stroke recovery, which is why stroke has a very high rate of disability. Targeted inhibition of pyroptosis may be a viable strategy for the clinical treatment of stroke, and many targeted inhibitors of the pyroptosis pathway have shown good inhibitory effects. The use of both NLRP3 and Caspase-1-targeted inhibitors was effective in improving stroke symptoms, which was associated with inhibition of the pyroptosis pathway. In addition, many TCMs have shown good potential in the treatment of stroke. GSDMD is a downstream physiological substrate of the scorch death pathway and its targeted inhibitors are of great interest. DMF, NAS, and disulfiram are drugs approved by the FDA for the treatment of other diseases. They are all direct inhibitors of GSDMD, preventing the formation of pores by binding to the Cys^191^/Cys^192^ of GSDMD, thereby reducing pyroptosis [89–92]. Pyroptosis is not the only form of death experienced by cells in the brain tissue during stroke, and its effect on stroke tends to be stronger in the early stages. Targeted inhibition of pyroptosis may improve symptoms in patients with stroke by increased the treatment time window and reducing neuronal damage. Reducing neuronal cell damage and recovering neuronal cells after stroke represent the central challenge in stroke treatment, with pyroptosis as a potential key target.

## Data Availability

Not applicable.
